# The pragmatic, rapid, and iterative dissemination and implementation (PRIDI) cycle: adapting to the dynamic nature of public health emergencies (and beyond)

**DOI:** 10.1186/s12961-021-00764-4

**Published:** 2021-08-04

**Authors:** Reza Yousefi Nooraie, Rachel C. Shelton, Kevin Fiscella, Bethany M. Kwan, James M. McMahon

**Affiliations:** 1 Department of Public Health Sciences, University of Rochester, Rochester, NY USA; 2 Institute of Health Policy, Management, and Evaluation, University of Toronto, Toronto, Canada; 3 Department of Sociomedical Sciences, Columbia University, New York, NY USA; 4 Department of Family Medicine, University of Rochester, Rochester, NY USA; 5 Department of Family Medicine, University of Colorado Anschutz Medical Campus, Aurora, CO USA; 6 School of Nursing, University of Rochester, Rochester, NY USA

**Keywords:** Rapid cycle, Public health emergencies, D&I models, COVID-19

## Abstract

**Background:**

Public health emergencies—such as the 2020 COVID-19 pandemic—accelerate the need for both evidence generation and rapid dissemination and implementation (D&I) of evidence where it is most needed. In this paper, we reflect on how D&I frameworks and methods can be pragmatic (i.e., relevant to real-world context) tools for rapid and iterative planning, implementation, evaluation, and dissemination of evidence to address public health emergencies.

**The pragmatic, rapid, and iterative D&I (PRIDI) cycle:**

The PRIDI cycle is based on a “double-loop” learning process that recognizes the need for responsiveness and iterative adaptation of implementation cycle (inner loop) to the moving landscapes, presented by the outer loops of emerging goals and desired outcomes, emerging interventions and D&I strategies, evolving evidence, and emerging characteristics and needs of individuals and contexts. Stakeholders iteratively evaluate these surrounding landscapes of implementation, and reconsider implementation plans and activities.

**Conclusion:**

Even when the health system priority is provision of the best care to the individuals in need, and scientists are focused on development of effective diagnostic and therapeutic technologies, planning for D&I is critical. Without a flexible and adaptive process of D&I, which is responsive to emerging evidence generation cycles, and closely connected to the needs and priorities of stakeholders and target users through engagement and feedback, the interventions to mitigate public health emergencies (e.g., COVID-19 pandemic), and other emerging issues, will have limited reach and impact on populations that would most benefit. The PRIDI cycle is intended to provide a pragmatic approach to support planning for D&I throughout the evidence generation and usage processes.

## Background

Public health emergencies—such as the 2020 COVID-19 pandemic—dramatically accelerate the need for evidence generation and synthesis, as well as the rapid dissemination and implementation (D&I) of evidence-based practices and interventions [1–3]. In a matter of weeks in late winter 2020, the scientific enterprise in clinical and translational research in public health and medicine was nearly universally reoriented to pressing and emergent COVID-19-related concerns. In addition to research on tests and treatments, there is a need for studying emerging healthcare system-level interventions. From the rapid adoption of telehealth services across nearly every health discipline [4–6], to the development and implementation of procedures for risk stratification and delaying elective procedures during the pandemic [7], to strategies for reopening and revamping healthcare and messaging considering physical distancing principles [8, 9], the pandemic became a driving force for rapid change in healthcare and public health systems.

D&I science has emerged as an evolving field to address the well-documented gap between research and practice [10]. Dissemination specifically relates to the active or planned communication of best practices and evidence-based interventions to encourage their widespread adoption among key decision-makers across a range of settings, whereas implementation focuses on factors and strategies to support the adoption and the routine use and delivery of the recommended practices or evidence-based interventions in real-world clinical and community settings [11]. The D&I of organizational- and system-level interventions, practices, or policies often involve modifying existing structures (physical or technological), redesigning processes of work and clinical workflow, and redefining roles, operating within a broader complex and dynamic organizational or healthcare system context. In emergencies, these already challenging modifications can become even more burdened by strained resources, competing demands, and overextended or strained systems and healthcare workers. Decision-makers may think of systematic planning for D&I as expendable or irrelevant in emergencies—perhaps perceived as an academic exercise or too time-consuming with limited added value. However, unsuccessful or inequitable implementation of resource-intensive system-level interventions can result in treatment delays, inequities in access to and delivery of care, and poor population health outcomes—including death. For example, the inequitable delivery of care may partially explain the racial and ethnic disparities in COVID-19 mortality [12, 13], or delays in provision of diagnostic tests may reduce the effectiveness of contact-tracing strategies [14].

However, classical D&I frameworks and approaches may need rapid adaptations to be pragmatic and of use in rapidly evolving emergency situations. An important feature of emergencies is the quick, dynamic, and unpredictable course of events and evolving nature of science [15], which makes planning for D&I challenging. The following are some examples:

- The *health or healthcare problem* itself may be dynamic and rapidly changing. In February 2020, the main concern of many health systems was implementing case finding and quarantine strategies; in March 2020, it was allocating intensive care unit (ICU) beds and ventilators; in May 2020, safe strategies to gradually lifting lockdowns [16]; in early 2021, how to administer mass vaccination; and in summer 2021, how to address the significant vaccine hesitancy [17]. This dynamic evolution of the problem affects the contexts of the study, selection of interventions and implementation strategies, and evaluation frameworks.
- The *evidence* and associated interventions or solutions and strategies to support delivery of evidence are not fixed, as the evidence for effectiveness of cloth masks, hydroxychloroquine, antibody tests, and various diagnostic approaches has evolved rapidly [18]. The COVID-19 “infodemic” [19] resulted in the outpouring of misinformation, which complicated the separation of fact from fiction and contributed to confusion in messaging among the public, as well as erosion of public trust in the information provided.
- The *contexts/settings* in which COVID-19 is being transmitted and in which testing, vaccination, and treatment occur are dynamic. Both inner settings (hospital resources, hospital policies, capacity, exhaustion) [20, 21] and outer settings (effective social distancing, vaccination rates, economic constraints, state/national policies) [22, 23] are changing by the day and over time, and require continuous monitoring and reconsideration of plans.
- *Key stakeholders (e.g., healthcare workers, patients, community members, leadership)* within systems and broader communities have evolving concerns, needs, and values. Their readiness, knowledge, and capabilities are evolving based on changing circumstances and contexts; and these stakeholders’ trust in medical institutions and perceptions of the importance of scientific evidence varies.
- There is usually *redundancy* and *parallelism* within systems, which positively and negatively affects the implementation of evidence-based processes and practices. On the positive side, we can learn from the experience of other health systems who deal with similar situations and challenges (e.g., in allocating ventilators and ICU beds) [24]. On the negative side, redundancy and parallelism and lack of communication may result in confusion, conflicts, dilution of resources, burn-out, and lack of monitoring and evaluation of what practices are both feasible and have impact.
- Additional complexities and considerations that need to be addressed include the striking racial/ethnic inequities that have been apparent with respect to COVID-19 morbidity and mortality, related in part to embedded systems that create and reinforce structural and interpersonal forms of inequity, discrimination, and racism [13, 25].

To align the science of D&I with the practice of D&I in real-world settings, it is important to explicate how health systems can apply D&I frameworks and methods rapidly, effectively, equitably, and with few resources to guide local adoption of evidence-based interventions or emerging best practices/protocols (informed by the best available evidence at the time). In this paper, we reflect on widely adopted D&I frameworks and tools and how they can be adapted to address dynamic trajectories of public health emergencies.

## The pragmatic, rapid, and iterative D&I (PRIDI) cycle

**Fig. 1:**
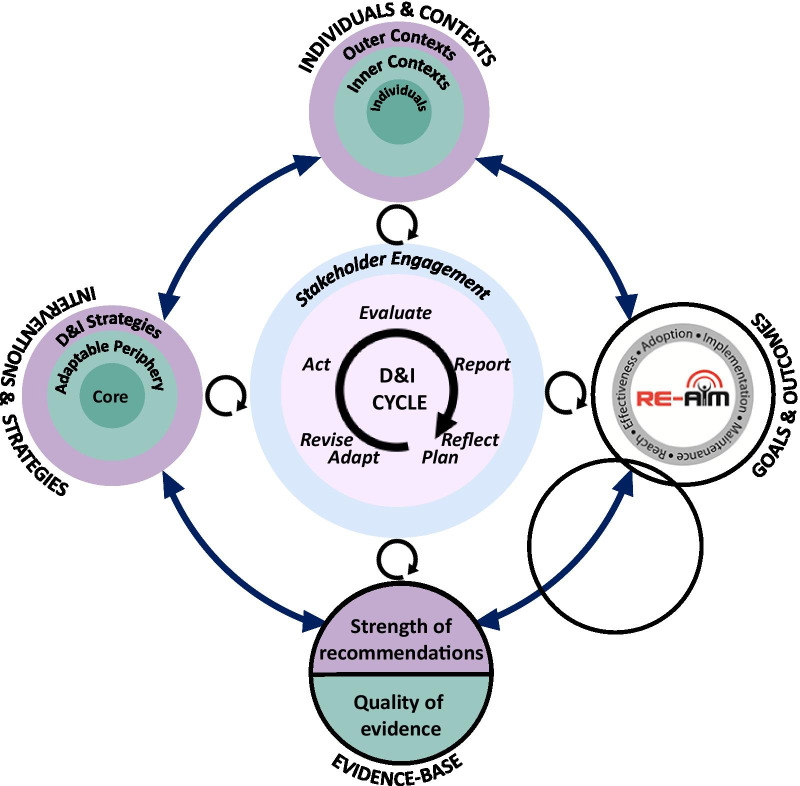
The PRIDI cycle

Figure 1 shows the PRIDI model for D&I. It depicts the dynamic connection between the cyclical process of executing and evaluating D&I (centre), the interventions and strategies (left side), the evolving nature of evidence (bottom), the multilevel nature of the context (upper side), and goals and outcomes of D&I (right side). Consistent with recent emphasis on the iterative and pragmatic nature of D&I [26, 27], the implementation journey is not a linear process, particularly in the fluid and dynamic contexts of emergencies. This cyclical process of Assess > Plan > Do > Evaluate > Report should be done rapidly and iteratively as an intervention and strategies to support its implementation are rolled out [28], a process that highlights the overlap between D&I and quality improvement approaches [29].

As shown in Fig. 1, the cyclical process resides at the centre of the PRIDI model. The inner circle involves the cycle of D&I, which activates and is influenced by the outer circle that involves revisiting the mental models, goals and outcomes, interventions and D&I strategies, and individuals and contexts through the course of D&I. It resembles a double-loop learning model [30, 31]. While single-loop learning involves incremental corrective actions aiming to improve current processes, and are most suitable for more stable conditions and contexts, we argue that the dynamic and evolving nature of emergencies calls for more complex learning processes and rapid refinements. If we apply Plan > Do > Study > Act (PDSA) cycles using existing models (i.e., single-loop learning), we might fail to learn from the higher-order feedback loops that require more than incremental improvements in efficiency and time [32]. Second-order learning might inform entirely different approaches based on different assumptions and different mental models. This mindset could even be extended to triple-loop learning (meta-learning, i.e., learning about learning) through which the process of reflection and learning is adapted in response to emerging complexities [33], considering the scarcity of time and resources.

To the extent possible, monitoring and iterative evaluation should be prioritized, and results should be regularly communicated and interpreted in partnership with stakeholders, and meaningfully and consistently incorporated in any redesign or planned adaptations/modifications within the system [2]. If an intervention or a D&I strategy is ineffective (or worse, proves harmful), it should be modified or abandoned (de-implemented) in a timely manner. Evaluations and monitoring may include information that changes the nature of the evidence supporting the effectiveness of the intervention itself or strategies to support its use (see cyclical path from the implementation to intervention and strategies).

The engagement of stakeholders within these dynamic contexts is critical throughout this process to understand what is working or not and why, where inequities are emerging, and the feasibility and acceptability of the programmes and practices. The double-loop nature of the process also has implications for engagement of diverse stakeholders and providing opportunities where people feel free to express contrarian views, thus challenge conventional assumptions. For example, suppose we assume that African Americans by virtue of higher SARS-CoV-2 infection rates and worse COVID-19 outcomes should be prioritized for testing, vaccination, and treatments. This would be a reasonable assumption from which we could develop cyclical PDSA strategies for messaging regarding testing, vaccination, and treatment. This assumption suggests that finding ways to promote awareness and access in the African American community regarding where to get tested and where to receive vaccines and treatments will reduce disparities in infection. Yet, if the African American community were at the table and divergent views were encouraged based on recognition of second-order learning, members might express reservations about COVID-19 testing, vaccination, and treatment, including risks related to family separation, forced quarantine without pay, and greater stigma. Similarly, members might voice deep scepticism towards receiving vaccines, including mistrust of government statements, concerns about a vaccine that has been rushed to market, and/or concerns about the vaccine safety, or treatments perceived as expensive, inaccessible, or unsafe. This second-order learning might suggest a fundamentally different approach from that of incremental changes in content, dose, or frequency of messages.

In Table 1, we summarize the suggested information that should be collected, discussed, and re-evaluated at each round of PRIDI cycle

**Table 1:** A template for recording progress in PRIDI cycles

Rounds		D&I goals	Intervention/evidence	Intervention Adaptations/refinements	Individuals: users, D&I actors	Settings (inner and outer context)	D&I strategies	Other key stakeholders	D&I/effectiveness evaluation Metrics (RE-AIM domains) with a focus on equity
Round 1	Description								
	Opportunities and challenges								
	Plans for next round/plans to address challenges								
Round 2	Progress/ Adaptations/revisions								
	Opportunities and challenges								
	Plans for next round/plans to address challenges								
Round 3 and up	Iteration of activities at Round 2								

## Interventions and strategies

Consistent with the RE-AIM [reach, effectiveness, adoption, implementation, and maintenance]/PRISM [Practical, Robust Implementation and Sustainability Model] framework [34], the interventions and strategies to facilitate their dissemination, adoption, and use are the key elements of D&I efforts, which are shown on the left side of Fig. 1:

- The *intervention (e.g., evidence-based practice, policy, programme, treatment)* to be disseminated and implemented (e.g., COVID-19 vaccines, diagnostic tests, and potential treatments, setting up an online meeting model for grand rounds, best practices for mental health screening among COVID-19 patients/healthcare workers, safety protocols and policies for birthing mothers) (e.g., the “what”)
- *D&I strategies* involve the processes, approaches, or interventions that facilitate and enhance the proactive D&I of the interventions. Examples include tailored email/online communication for the self-assessment platform, literacy-appropriate instructional packages for patients about the COVID-19, staff training/education to learn about the new workflow, and motivational incentives to enhance staff participation in grand rounds. See Powell et al. (2015) for a taxonomy of implementation strategies based on the Expert Recommendations for Implementing Change (ERIC) project [35].

Interventions generally include a core (the essence or function of the intervention that is responsible for its impact) and an adaptable periphery (that could be modified to adapt to various contexts and situations) [36]. Ignoring the distinction of these two components may result in rigid interventions that are not sufficiently flexible to survive varying and unprecedented contextual variations and barriers, or that are too complex or costly to be implemented. As such, it is important that a flexible approach is taken during the design of D&I activities, and the local implementers are trusted to adapt the intervention to fit into their own local contexts, resources, needs, and policies. Consequently, we added adaptation as an important phase in the PRIDI cycle (centre of Fig. 1).

D&I adaptation models may be useful to help guide planned adaptations (e.g., ADAPT-ITT [assessment, decision, administration, production, topical experts, integration, training, testing]) [37], to help balance considerations of fit and fidelity. Ideally, the core component of the intervention should be defined, dynamically updated (as changes are made over time), and communicated; relevant data could be collected through iterative evaluations to understand the impact of both the core elements of the programme and any planned adaptations made, as well as the evolution of the programme across its life course [38]. For example, preventive health messages delivered through health organizations such as the Centers for Disease Control and Prevention (CDC) [39] and local and state health authorities typically target broad audiences and are not always adapted to the needs, values, or expectations of vulnerable individuals and communities. The messages may not address the limited behavioural control of the target audience (e.g., in practising social distancing or staying at home), may not include information about local services and resources, and may not be adapted to the literacy levels of individuals who may be at greatest risk for COVID-19 [40, 41]. For example, an individual living in a dense, multigenerational household may have difficulty adhering to isolation and physical distancing guidelines or may lack digital technology to access electronic health literacy resources [42, 43]. Communities of colour, including Black Americans who have experienced striking COVID-19 inequities, are more likely to be exposed to multiple layers of structural racism, including living in buildings and neighbourhoods that are more crowded and have poorer infrastructures, irrespective of income, and may feel unsafe using face masks in public. Asian or Asian American groups may face stigma, discrimination, and violence related to the disease due to misinformation about its origins and spread. An individual living in unstable economic conditions who needs to work may not be able to self-isolate for the recommended period while symptomatic. Therefore, standard messaging should be adapted to the needs, expectations, and capacities of diverse subgroups and populations to be able to educate or motivate and improve the understanding of COVID-19 and both individual and community responses to it.

## Evidence base

COVID-19 is a great example of the importance of implementing solutions as their evidence base is continuously evolving over time. This includes the evidence supporting diagnostic tests and therapeutic interventions, systemic interventions (such as lockdowns and public mask use, vaccination strategies, and awareness of and access to evidence-based treatments), and social issues and their corresponding interventions (such as approaches to address vaccine hesitancy). Such interventions are being implemented while their evidence base is limited and evolving [44]. Under time constraints and public pressure, decision-makers feel an urgent need to make prompt and clear decisions that are acceptable to the public and based in science. But assessing the quality of evidence and crafting careful recommendations are as critical in emergencies as in usual practice. For example, the Grading of Recommendations Assessment, Development and Evaluation (GRADE) approach for assessing the quality of evidence and formulating recommendations, which has been widely used in guideline and policy development [45], has been adapted to emergencies and shorter time frames to address the need for evidence for effectiveness of interventions to address COVID-19 [46].

The experience of COVID-19 boosted efforts to revise the traditional evidence pipeline to be more responsive to dynamic changes [47], and to attend to social aspects of evidence generation such as equity, acceptability, and feasibility [48]. Depending on the specific health or healthcare issue and its corresponding interventions, the nature of the evidence and indicators of evidence quality may differ. While high-quality academic research is the broadly accepted source of evidence, many public health and social issues require a broader definition of evidence, including local evaluations, policy documents, population-based data, community-defined evidence, and professional experience [49]. For example, the challenges to mask use and vaccine hesitancy, and locally acceptable solutions to address those challenges, could best be addressed using localized and culturally tailored surveys, qualitative interviews, and focus groups, as well as surveying comparable subgroups in regularly collected surveillance data and polls. Recognizing the shortcomings of existing surveillance systems in response to emergencies such as COVID-19, and the development of dynamic, adaptable, and responsive data infrastructure and mechanisms have been recognized as a health system priority.

## Goals and outcomes

Evaluation is not a one-time post-intervention process in D&I; it is an iterative, ongoing process that can enhance and inform the evolvability of evidence-based interventions and strategies, including their design, adaptation, refinement, and delivery throughout the process of D&I. Consequently, intended goals and outcomes of D&I should ideally be incorporated from the beginning (right side of Fig. 1). In emergency planning, the value of learning from continuous evaluation is even more essential, as the path forward can be more uncertain, the interventions are more experimental and their evidence-base evolving, and the clinical situation and healthcare contexts can change quickly. As such, it may be useful for decision-makers to have a compass to guide them as to whether they are moving in the right direction or need to reassess and redesign and challenge existing models that might not fit with such a dynamic context.

RE-AIM provides a systematic conceptual framework to guide the planning, adaptation, and evaluation of the D&I activities, programmes, practices, or policies [26, 50]. An intervention should *reach* the target populations equitably (Did we reach the those who needed the intervention or would benefit the most from it?); be *effective* (Did the intervention achieve its goals and impact on health behaviours/outcomes?); be widely *adopted* (Did the settings and stakeholders/decision-makers adopt the intervention?); be *implemented* (Did the target users or implementers actually use it as it was intended? How was it adapted?); and be *maintained*/*sustained* (Did the target users continue using it over time and did it continue to have long-term impact?). Importantly, in light of dynamic contexts [51], RE-AIM can be iteratively applied to track these D&I indicators to help document where inequities and challenges in each of these areas are arising and to inform refinements of adaptations to respond to changing system challenges (e.g., costs, resources), population needs/values, and evolving evidence [26, 52].

Glasgow et al. (2020) applied RE-AIM iteratively in a participatory process to support prospective adjustments during implementation projects [27]. Through this cyclic process, it may be useful for implementing agents/teams to receive practical and customized feedback about their performance, so they can understand progress in comparison to the original goals or in comparison to other implementers in their setting, and correct their path if needed [53]. RE-AIM dimensions may differ in terms of importance and feasibility of assessment. At each round of the cycle, stakeholders can decide which RE-AIM dimensions are more important, more in need of improvement, and are potentially more feasible to assess [27, 52].

## Individuals and contexts

The upper side of Fig. 1 shows the multilayered and complex nature of contextual factors and their role in determining the success or failure of D&I efforts. It is critical to consciously consider the complexity of personal, interpersonal, organizational, social, economic, policy, community, and cultural contexts at the design phase, and across the continuous process of re-evaluation and adaptations throughout implementation phases. A seemingly useful intervention may fail to be realized, since patients may find it irrelevant to their needs and characteristics, or may face certain financial and structural/logistical barriers to accessing and using it, or may not trust the source of the intervention; staff or administrators may find it burdensome (since many staff who are running these programmes are delivering them in addition to their normal workload, they may be overwhelmed or have many competing demands under limited resources); and at the organizational level, infrastructure needed to deliver the programme may have geographical, demographic, and structural limitations. External environment factors such as policies, economic challenges, and cultural and social norms are also rapidly changing. For example, adherence to long-term physical/social distancing may vary based on demographics and cultural backgrounds [54]; country-level and state-level disease mitigation policies may affect the implementation and sustainment of interventions [55]; and wider economic impact of the lockdowns and current mitigation strategies may affect the effectiveness and sustainment implementation of those mitigation strategies (through activation of feedback loops) [56]. Many of these barriers are difficult to overcome in emergency situations; however, having the tools to recognize and address them may facilitate development of innovative alternative solutions and enhance the reach and impact of evidence-based intervention, particularly with prioritization on health equity.

## Stakeholder engagement

It may seem like an inconvenient time to engage stakeholders in the context of emergency situations. However, even brief engagement of stakeholders has immense benefits that make it worth prioritizing, at the design phase and through the cyclical process of re-evaluation and redesign [57]. Stakeholders that are actively involved and engaged in the processes of D&I may [58]:

- feel more invested to help disseminate, implement, and sustain an intervention or public health practice;
- be prepared cognitively and operationally and be more committed to execute plans for adoption of an intervention or public health practice;
- identify setting- or cultural-specific barriers that may have been have missed;
- provide real-time feedback on whether strategies are working and inform important refinements or adaptations of interventions and strategies; and
- enhance relevance and fit, and may propose innovative solutions.

Stakeholder engagement may be applied at different degrees along the spectrum of implementation, depending on the availability of time and resources and the nature of the intervention and D&I strategies [59]. Even at the most basic levels of engagement (i.e., information provision and consultation), involving stakeholders in planning, dissemination, and interpretation and sense-making can facilitate preparedness and elicitation of feedback critical in the success of D&I efforts. Given its importance in informing and guiding the process of D&I, “stakeholder engagement” is shown as a circle surrounding all phases of the D&I cycle in the PRIDI model (Fig. 1).

## Leadership

All mentioned processes are only possible under the context of strong organizational commitment [60], as well as transformational (inspiring and motivating) and transactional (providing contingent rewards) leadership [61], that have shown to predict implementation success [62]. Organizational leaders can help maximize the fit between all aspects of D&I activities [34], make and effectively communicate strategic decisions, and are nimble and ready to change course midway if the iterative evaluations suggest the need for modification of goals and strategies. A successful crisis leader should be well-versed with the subject matter (e.g., public health) or consult team members with expertise in the specific area; should make evidence-based and timely decisions, while continuously collecting data from the environment; should inspire trust and confidence; and should feel responsible for the safety and welfare of the team members [63]. In emergency situations, it is very likely that multiple groups try independently to develop solutions, which may result in fragmented efforts and confusion. The leader should develop an effective project management structure as well as an atmosphere in which teams and individuals have the means and feel free to express criticisms and suggest alternative solutions. Finally, the leader should highlight the importance of and provide resources necessary to apply the processes and principles central to the PRIDI framework.

## Conclusions

In this paper, we reflected on the cyclical model of Assess > Plan > Do > Evaluate > Report [28], the RE-AIM/PRISM framework [34], and recent advancement of RE-AIM to incorporate equity [26] and to inform rapid implementation [27]. We proposed the PRIDI model that takes the dynamic nature of problems, interventions, evidence, contexts, and stakeholders into account. D&I in the context of emergency should be a continuous and iterative process. RE-AIM provides a framework for the evaluation of D&I activities, that includes *reach, effectiveness, adoption, implementation*, *maintenance*. Recent extensions of this model can also inform more explicit consideration of understanding and addressing health equity and equitable implementation over time and in dynamic contexts [26]. Interventions are disseminated and implemented in complex and multilayered contexts. Overlooking these complexities will hamper the success of the adoption, use, and impact of the intervention.

The cyclical process of D&I informs double-loop learning processes that may result in revisiting mental models, goals and outcomes, interventions and D&I strategies, and individuals and contexts. The results of cyclical evaluations should also be communicated with local implementers and stakeholders through customized and actionable feedback. Stakeholder engagement is a key solution to understand and address contextual variations and barriers. It is a continuum ranging from informing the stakeholders to co-ownership, and will be critical to addressing some of the striking racial/ethnic and setting inequities evidenced for COVID-19, including redistribution of decision-making and resources with the community. Learning from and with communities is broadly recognized as an important source of evidence to guide learning organizations and health systems [64].

During an epidemic, the priority of the health system is provision of evidence-based prevention and treatment, while the priority of the research community is rapid development of effective diagnostic and therapeutic technologies. Even though the health system priority at this moment is the provision of the best care to the individuals in need and the development of effective diagnostic and therapeutic technologies [2], prospective, flexible D&I planning is also critical [2, 65]. Without planning and tailoring, meaningful partnerships, and engagement of local stakeholders, D&I strategies will never reach target populations that would most benefit, but rather will be primarily accessed and used by sociodemographic groups that face fewer structural barriers to care (hence deepening the equity gap); and will not sustain as intended. While limited organizational readiness and lack of time and resources are challenges to effective D&I plans, emergency response interventions may fail to meet their objectives and waste limited resources if critical D&I considerations are ignored.

Key to preparing for national emergencies such as COVID-19 are the development of infrastructures, organizational cultures, trainings, and establishment of processes towards a rapid-learning health system (LHS) [66, 67] that is grounded in D&I as its key component [67]. These steps will prepare healthcare systems and organizations to effectively respond to future emergencies. An LHS, as a type of learning organization, develops capacities for both single-loop and double-loop learning at the individual and organizational levels [64]. An LHS paradigm facilitates the processes of evidence generation and synthesis through the development of interoperable data platforms and infrastructure to provide real-time and adaptable data to continuously inform policies and practices [68]. The iterative process of data aggregation, analysis, interpretation, feedback, and change is responsive to the emergent nature of evidence and the need for learning from and with stakeholders, including communities and frontline practitioners. COVID-19 underscores the importance of accelerating progress towards creation of genuine LHS [68, 69].

This paper calls for dynamic and adaptive D&I models that are responsive to the rapid and unpredictable nature of emergencies through a double-loop process (or triple-loop, considering time, resources, and complexity) involving rapid and iterative cycles of implementation through continuous engagement of stakeholders that are embedded in and adapted for the emergent and evolving nature of goals, interventions, evidence base, and contexts. Establishing these models is essential to preparing for the next national health crisis.

## Data Availability

Data sharing is not applicable to this article as no data sets were generated or analysed during the current study.

## Abbreviations

CDC: Centers for Disease Control and Prevention
D&I: Dissemination and implementation
ERIC: Expert recommendations for implementing change
PDSA: Plan, do, study, act
PRIDI: Pragmatic, rapid, and iterative dissemination and implementation
PRISM: Practical, Robust Implementation and Sustainability Model
RE-AIM: Reach, effectiveness, adoption, implementation, and maintenance
