# Correction to: The pragmatic, rapid, and iterative dissemination and implementation (PRIDI) cycle: adapting to the dynamic nature of public health emergencies (and beyond)

**DOI:** 10.1186/s12961-021-00771-5

**Published:** 2021-08-19

**Authors:** Reza Yousefi Nooraie, Rachel C. Shelton, Kevin Fiscella, Bethany M. Kwan, James M. McMahon

**Affiliations:** 1 Department of Public Health Sciences, University of Rochester, Rochester, NY USA; 2 Institute of Health Policy, Management, and Evaluation, University of Toronto, Toronto, Canada; 3 Department of Sociomedical Sciences, Columbia University, New York, NY USA; 4 Department of Family Medicine, University of Rochester, Rochester, NY USA; 5 Department of Family Medicine, University of Colorado Anschutz Medical Campus, Aurora, CO USA; 6 School of Nursing, University of Rochester, Rochester, NY USA

## Correction to: Health Res Policy Sys (2021) 19:110 10.1186/s12961-021-00764-4

Following the publication of the original article [1], we were notified that an out-of-place circle had been mistakenly included in Fig. 1, without any impact on the scientific content of the figure.

The original article has now been corrected.
